# Yang cycle enzyme DEP1: its moonlighting functions in PSI and ROS production during leaf senescence

**DOI:** 10.1186/s43897-022-00031-2

**Published:** 2022-04-20

**Authors:** Chu-Kun Wang, Xiu-Ming Li, Fang Dong, Cui-Hui Sun, Wen-Li Lu, Da-Gang Hu

**Affiliations:** 1 National Key Laboratory of Crop Biology; MOA Key Laboratory of Horticultural Crop Biology and Germplasm Innovation; College of Horticulture Science and Engineering, Shandong Agricultural University, Tai’an, 271018 Shandong China; 2 Shandong Institute of Pomology, Key Laboratory for Fruit Biotechnology Breeding of Shandong, Tai’an, 271000 Shandong China

**Keywords:** DEHYDRATASE-ENOLASE-PHOSPHATASE-COMPLEX1, Yang cycle, Photosystem I, ROS, Leaf senescence, Photosynthesis

## Abstract

Ethylene-mediated leaf senescence and the compromise of photosynthesis are closely associated but the underlying molecular mechanism is a mystery. Here we reported that apple DEHYDRATASE-ENOLASE-PHOSPHATASE-COMPLEX1 (MdDEP1), initially characterized to its enzymatic function in the recycling of the ethylene precursor SAM, plays a role in the regulation of photosystem I (PSI) activity, activating reactive oxygen species (ROS) homeostasis, and negatively regulating the leaf senescence. A series of Y2H, Pull-down, CO-IP and Cell-free degradation biochemical assays showed that MdDEP1 directly interacts with and dephosphorylates the nucleus-encoded thylakoid protein MdY3IP1, leading to the destabilization of MdY3IP1, reduction of the PSI activity, and the overproduction of ROS in plant cells. These findings elucidate a novel mechanism that the two pathways intersect at MdDEP1 due to its moonlighting role in destabilizing MdY3IP1, and synchronize ethylene-mediated leaf senescence and the compromise of photosynthesis.

**Supplementary Information:**

The online version contains supplementary material available at 10.1186/s43897-022-00031-2.

## Core

Yang cycle enzyme MdDEP1 interacts directly with and dephosphorylates the nucleus-encoded thylakoid protein MdY3IP1, leading to the destabilization of MdY3IP1, the reduction of the PSI activity, and the overproduction of ROS during leaf senescence. The finding shed light on the current understanding of the roles of the moonlighting enzyme MdDEP1 in modulation of photosystems and leaf senescence.

## Gene & Accession Numbers

Sequence data from this article can be found in the Genome Database for Rosaceae (https://www.rosaceae.org/) under accession numbers *MdDEP1* (MDP0000180420), *MdY3IP1* (MDP0000930948).

## Introduction

Photosynthesis effectively utilizes sunlight to convert carbon dioxide into useful biomass and underpins the survival of virtually all higher life forms on earth (Nelson and Ben-Shem, 2004). As the primary processes of photosynthesis, photosynthetic light reactions are driven by the four, multi-subunit, thylakoid membrane-embedded pigment-protein complexes that are known as the photosystem I (PSI), the photosystem II (PSII), the cytochrome (Cyt) *b*_*6*_*f* complex, and the F-ATPase. These complexes together with mobile electron carriers plastoquinone and plastocyanine drive photosynthetic electron transport through the thylakoid membranes of the chloroplasts (Nelson and Ben-Shem, 2004). Among them, PSI is an important pigment-binding protein complex that has been recognized as essential for oxygenic photosynthetic organisms. It drives electron transport from plastocyanin to ferredoxin by absorbing sunlight and utilization of its energy (Amunts et al. 2010; Wientjes and Croce 2011).

In higher plants, PSI comprises 12 to 19 polypeptide subunits, depending on the organism, that hold P700, four light-harvesting complexes (Lchca 1–4) and the reaction center (RC), and bind approximately two hundred pigments especially chlorophylls (Chls) and cofactors (Mazor et al. 2015; Qin et al. 2015). On the one hand, it is a source of reactive oxygen species (ROS) that is one of the main causes of photodamage (Vass, 2012). On the other hand, it can be protected against photodamage through PROTON GRADIENT REGULATION 5 (PGR5) protein-dependent processes and photoinhibition of PSII (Shikanai 2014; Tikkanen et al. 2014). Therefore, PSI has been considered as a very robust photosystem in plants. In addition, PSI is also resistant to high-light stress, and is generally damaged under specific conditions such as at a low temperature or in the presence of an active PSII, especially in the absence of the PGR5 protein (Scheller and Haldrup, 2005; Sonoike, 2011; Suorsa et al. 2012). There is no efficient repair mechanism for PSI, and thus its photoinhibition seems to be degradation of the whole PSI complex with that de novo biogenesis of the complex (Jensen et al. 2007; Sonoike 2011).

Although the light-harvesting chlorophyll protein complex I (LHCI) of PSI is closely related to the PSI core complex and forms the PSI-LHCI supercomplex, the PSI-LHCI assembly steps is still rather elusive. Nevertheless, several auxiliary proteins have recently been characterized to facilitate the biogenesis and assembly of PSI (Krech et al. 2012; Yang et al. 2015; Wang et al. 2016; Järvi et al. 2016; Shen et al. 2017). Among them, hypothetical chloroplast reading frame number 3 (Ycf3) and hypothetical chloroplast reading frame number 4 (Ycf4) are two plastid-localized proteins essential for the assembly of the PSI complex (Boudreau et al. 1997; Rochaix 2011a; Krech et al. 2012). Ycf3 protein also cooperates with the nucleus-encoded thylakoid protein Y3IP1 in PSI assembly of tobacco and *Arabidopsis* (Albus et al. 2010). Interestingly, more novel biosynthetic and assembly factors of PSI are recently discovered. For example, the thylakoid membrane-bound FtsH proteases are found to be responsible for the proper biosynthesis of PSI. Another thylakoid membrane protein PSA3 promotes the PSI accumulation through its interaction with the assembly factor PYG7 (Järvi et al. 2016; Shen et al. 2017). Additionally, it has been reported that PPD1 is associated with PSA2 and PYG7 to play critical roles in PSI biogenesis as well as PSI assembly (Stöckel et al. 2006; Liu et al. 2012; Fristedt et al. 2014; Wang et al. 2016).

Leaf senescence is a complex and highly-regulated event comprising the final stage of leaf development. It is critical for the fitness of plants as nutrient reallocation from senescing cells into reproducing seeds and actively growing tissues is achieved through this process (Quirino et al. 2000). In general, the most noticeable early changes associated with the leaf senescence occurs in the chloroplasts, including the decrease of the thylakoid membrane density and the reduction of PSI and PSII activities (Lu et al. 2002). Furthermore, the whole process of leaf senescence is accompanied by the degradation of a large amount of chloroplast proteins (Hörtensteiner and Feller, 2002). Cellular senescence is actually considered as a transdifferentiation rather than a cell death process as it can be experimentally reversed, and it is essential to maintain the viability of plant cells when senescence is initiated (Zavaleta-Mancera et al. 1999; Thomas et al. 2003; Sakuraba et al. 2012). The application of molecular biology techniques to study leaf senescence during the past decades has enabled the characterizations of various senescence mutants and senescence-associated genes (SAGs), which showed the nature of regulatory factors and its complicated molecular regulatory network underlying leaf senescence (Zhang and Zhou, 2013; Koyama, 2014; van Deursen, 2014; Schippers, 2015).

Leaf senescence is controlled by environmental cues such as light and temperature, as well as by developmental signals such as hormones, especially phytohormone ethylene (Rumeau et al. 2007; Eberhard et al. 2008; Rochaix 2011b). The biosynthesis of ethylene starts from S-adenosyl methionine (SAM), an activated form of methionine. SAM is then converted to 1-aminocyclopropane-1-carboxylic acid (ACC) by ACC synthase, and ACC is finally oxidized by ACC oxidase (ACO) to form ethylene (Wang et al. 2002; Xu and Zhang 2014). The final biosynthesized compounds include the polyamines and the phytohormone ethylene after a series of biochemical reactions (Miyazaki and Yang, 1987). Among those enzymes of ethylene biosynthesis, ACC synthase (ACS) is a rate-limiting enzyme (Kende 1993). Aminoethoxyvinylglycine (AVG) is a known inhibitor of ACS activity (Yu and Yang, 1979), which converts S-adenosine methionine (SAM) to 1-aminocyclopropane-1-carboxylic acid (ACC), as a direct precursor of ethylene (Adams and Yang 1979). In addition to the de novo biosynthesis, there is a methionine salvage pathway (Yang cycle) that also contributes to the production of ethylene. The Yang cycle plays important roles in maintaining the continued production of polyamines and ethylene. All enzymes and intermediates of methionine salvage pathway have been identified in plants (Wray and Abeles, 1995). Of these enzymes, the plant-specific enzyme DEP1 is a trifunctional enzyme that has dehydratase, enolase, and phosphatase activities and converts 5-methylthioribulose-1-P (MTRu-1-P) directly to the 1,2-dihydroxy-3-keto-5-methylthiopentene (DHKMP), the reciprocal third compound in this pathway (Pommerrenig et al. 2011). In apple, a dehydratase-enolase-phosphatase gene *MdDEP1* was identified by the cDNA-AFLP approach and found to be associated with the low acidity of fruit in apple (Yao et al. 2007). *MdDEP1* could enhance drought and salt resistance in *Arabidopsis* and exhibit an early flowering phenotype (Wang et al. 2019). MdbHLH3 can regulate the transcriptional activity of *MdDEP1* and thus regulate the senescence of apple leaves (Hu et al. 2020). However, the specific regulatory mechanism of MdDEP1 regulation on senescence is still unknown.

Methionine-ethylene-mediated leaf senescence initiates a signaling cascade leading to the reduction of photosynthetic performance including inhibition of PSI and PSII activities (Quirino et al. 2000; Lu et al. 2002; Ceusters and Van de Poel 2018). The decrease of photosynthetic performance also accelerates the leaf senescence in plants (Hensel et al. 1993; Murchie et al. 1999; Quirino et al. 2000). However, the exact mechanism mediating the complex network of the leaf senescence and the photosynthesis in plants remain elusive. Here, a novel function of the apple MdDEP1 in the regulation of PSI by directly dephosphorylating nucleus-encoded thylakoid protein MdY3IP1 was characterized. Subsequently, the moonlighting functions of MdDEP1 in PSI activity and ROS homeostasis during leaf senescence, as well as its application for controlling plant photosynthesis and development breeding programs, are discussed.

## Results

### MdDEP1 is involved in chloroplast development

**Fig. 1:**
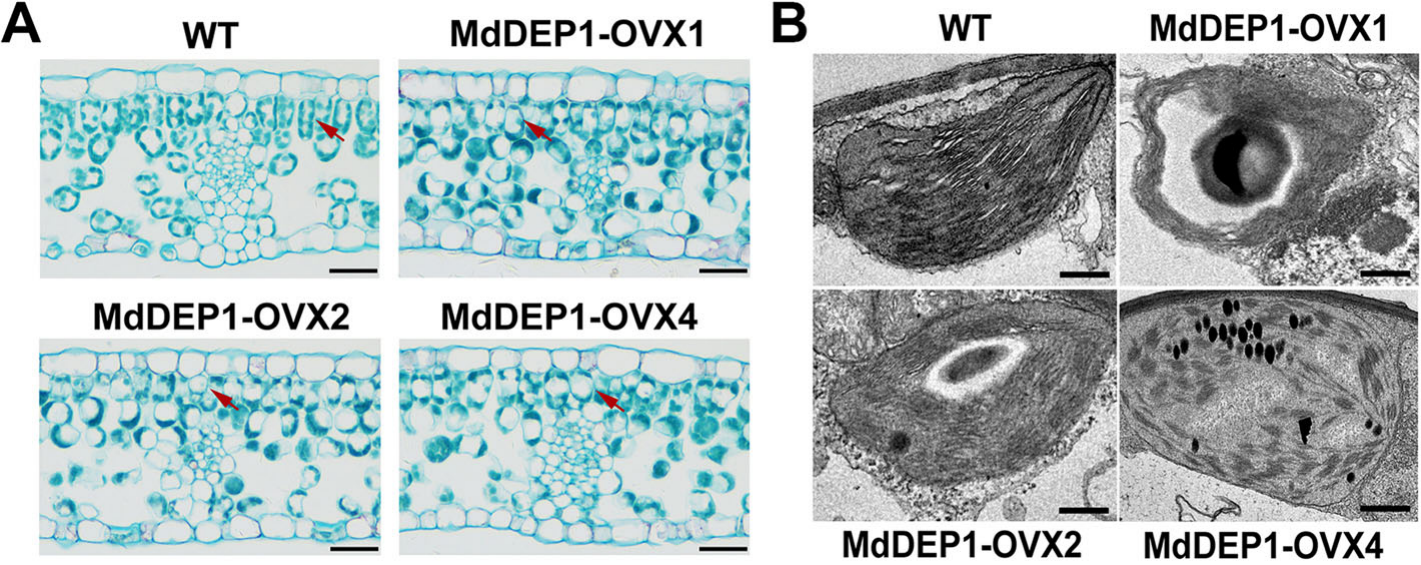
MdDEP1 plays key roles in chloroplast development. **A**. Light microscopy of leaves at T3 stage. The red arrows indicate the distribution of chloroplasts in mesophyll cells. Bars = 200 μm. **B**. Transmission electron microscopy of leaves at T3 stage. Bars = 2 μm

To determine the function of MdDEP1, we detected the expression level of MdDEP1 at different leaf stages in the same plant (Fig. S1A), and the results showed that the expression level of MdDEP1 was up-regulated with the increase of leaf senescence, suggesting that MdDEP1 may be involved in the plant senescence process. At the same time, previous studies have shown that *MdDEP1* could regulate the leaf senescence in apple (Hu et al. 2020) and the silencing of MdDEP1 by transient transformation can inhibit leaf senescence and chlorophyll degradation to a certain extent (Fig. S1B-D). To further explore the role of *MdDEP1* in senescence, the leaves of *MdDEP1* transgenic apple plantlets were observed by scanning electron microscopy. More details of the leaf morphology phenotypes, leaf regions exhibiting severe senescence phenotypes were sectioned and examined by light microscopy. A heterogeneous distribution of chloroplasts in mesophyll cells was observed from all three *MdDEP1*-overexpressing (MdDEP1-OVX1, −OVX2, −OVX4) plants but not in WT control (Fig. 1A). When observed under transmission electron microscope, the chloroplasts from these plants showed the typical phenotypes of non-appressed and disconnected stroma lamellae, as well as the swelling and abnormalities of thylakoid lumen compared to the WT (Fig. 1B). These data suggested that *MdDEP1* functions in leaf chloroplast development.

### MdDEP1 regulates the expression of genes functioning in the leaf senescence and the photosystems

**Fig. 2:**
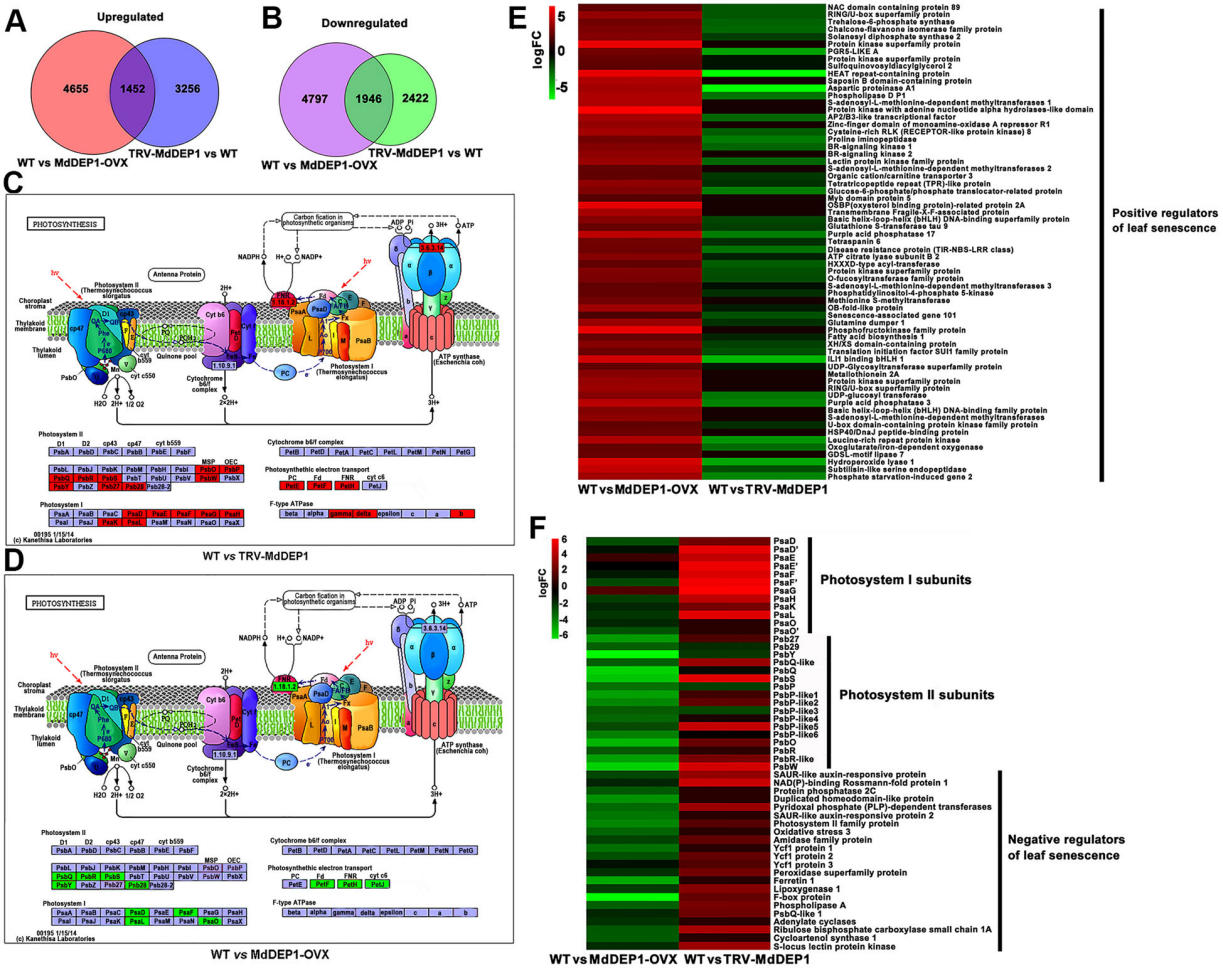
Gene expression profiling of *MdDEP1*-transgenic apple plantlets with RNAseq compared to the WT apple plantlets. **A-B**. Venn diagram analysis of common upregulated (**A**) and downregulated (**B**) genes (DEG; − 1.5 > logFC> 1.5; FDR < 0.01). **C-D**. Expression changes of genes involved in the photosynthetic pathway in WT vs TRV-MdDEP1 (**C**) and WT vs MdDEP1 (**D**), respectively. Red boxes, up-regulated genes; green boxes, down-regulated genes. **E-F**. Heat map showing selected differentially expressed genes with log2fold change scale. Means of three experiments are shown; complete data are given in Appendix S1 and S2. **E**. Positive regulator genes of leaf senescence. **F**. Negative regulator of leaf senescence and PSI and PSII genes

To explore the impact of *MdDEP1* overexpression at a whole-genome level, RNA-seq analysis were performed on leaves of the *MdDEP1*-overexpressing (MdDEP1-OVX), the *MdDEP1*-silencing (TRV-MdDEP1), and the WT apple plantlets. More than 6 Gb of sequence data was generated with about 35 million raw reads that averaged at about 120 bp. Subsequently, pairwise comparisons of transcript abundances were performed to identify differentially expressed genes [− 1.5 > logFC> 1.5; false discovery rate (FDR) < 0.01] comparing the WT and the three *MdDEP1*-overexpressing samples. A total of 12,850 genes were found to be differentially expressed in the *MdDEP1*-overexpressing plantlets compared to the WT (6107 up; 6743 down), whereas 9076 genes were found to be differentially expressed comparing the WT control to the *MdDEP1*-silencing plantlets (4708 up; 4368 down) (Fig. 2A and B; Appendix S1 and S2). Interestingly, among these differentially expressed genes, 1452 of them were up regulated and 1946 genes were down regulated in both *MdDEP1* overexpression and *MdDEP1* suppression plantlets (Fig. 2A and B).

To evaluate the global gene expression profiles, Gene Ontology (GO) enrichment analysis was performed on the genes with significant transcriptional changes in the above samples using InterPro and GO annotations. The annotations revealed that the absolute expression value (log2ratio) of *MdDEP1* in the MdDEP1-OVX apple plantlets compared to the WT was 7.46-fold, but 3.13-fold in the WT control compared to the TRV-MdDEP1 apple plantlets, based on an FDR < 0.01 (Appendix S1 and S2). Remarkably, the “Photosynthesis” KEGG pathway was one of the most enriched pathways in the RNA-seq data (Fig. 2C and D). A total of 63 differentially expressed genes were associated with the photosynthesis. Most of them were found to be significantly up-regulated in the TRV-MdDEP1 plantlets but down-regulated in the MdDEP1-OVX plantlets compared to the WT (Fig. 2C and D). Examples included the genes encoding the PSI subunits PsaD and PsaF and the PSII subunits PsbQ and PsbS, protein subunits essential for photosynthesis especially photochemical electron transfer (Fig. 2C and D). Furthermore, previous studies have shown that IIId subgroup bHLH factors bHLH03, bHLH13, bHLH14, bHLH17 and NAC transcription factors JUB1, AVNI2 negatively regulate leaf senescence, while IIIe subgroup bHLH TFs MYC2, MYC3, MYC4 and NAC transcription factors ANAC016, NAP, ORS1, ORE1 are positive regulators (Guo et al. 2021; Zou et al. 2021). A group of genes encoding these positive regulators of leaf senescence were up-regulated in the MdDEP1-OVX plantlets and down-regulated in the TRV-MdDEP1 plantlets, compared to the WT controls (Fig. 2E). To the contrary, a group of genes encoding the negative regulators of leaf senescence and the photosystem I, as well as some photosystem II subunits showed the opposite trend. These genes were up-regulated in the TRV-MdDEP1 plantlets but suppressed in the MdDEP1-OVX plantlets compared to the WT controls (Fig. 2F).

As a validation of the RNA-seq results, several genes encoding the photosystem I subunits and photosystem II subunits, as well as the leaf senescence regulators were analyzed with qRT-PCR. The results confirmed what was found in the RNA-seq analysis (Fig. S2A-C). Therefore, the alteration of *MdDEP1* expression seemed to not only cause leaf yellowing and impaired chloroplast development, but also induced a broad gene expression modification related to both leaf senescence and photosynthesis.

### *MdDEP1* overexpression has impacts on the Yang’s cycle

**Fig. 3:**
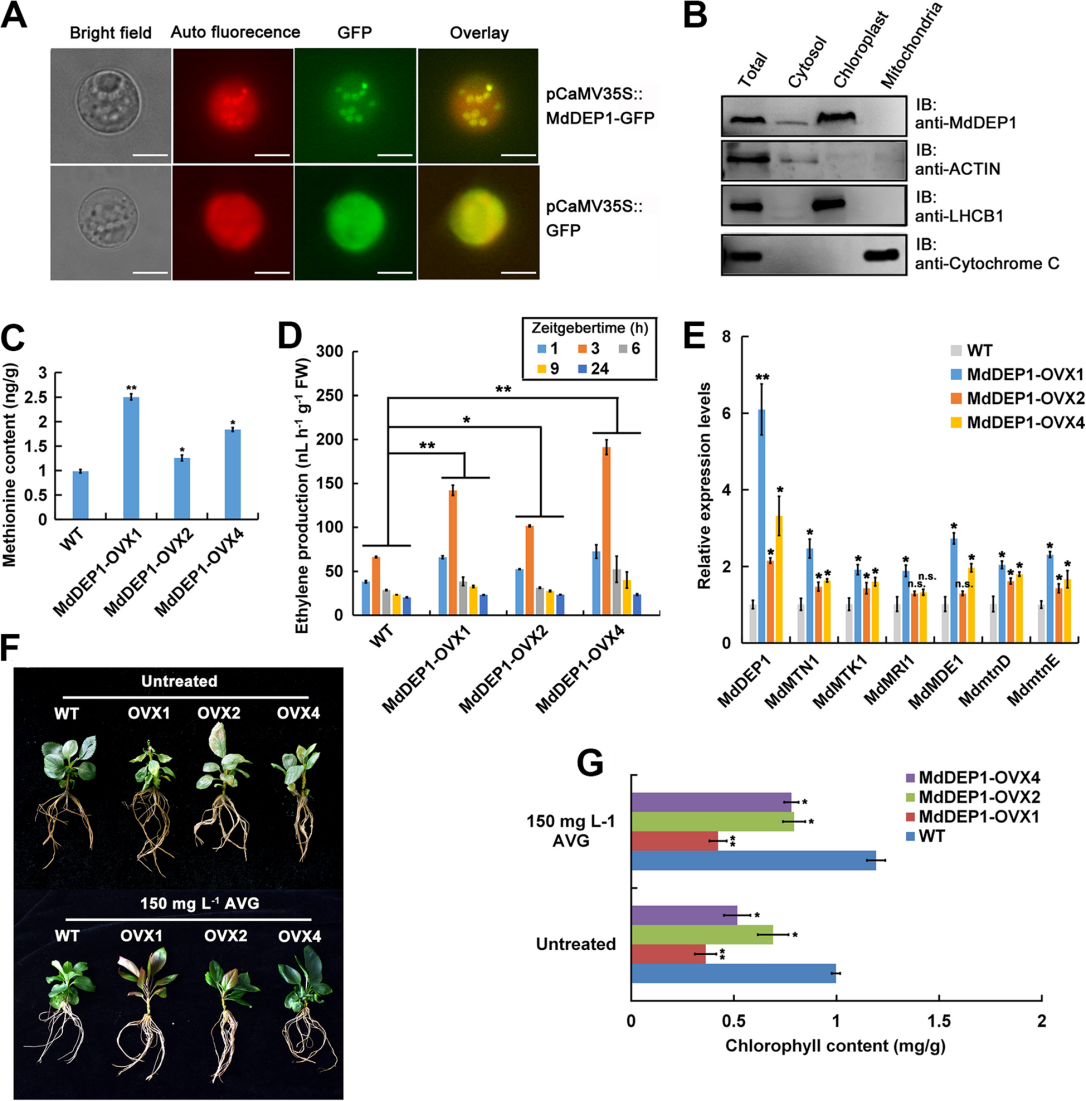
Effect of *MdDEP1* overexpression on the Yang’s cycle. **A**. Expression of *pCaMV35S::MdDEP1-GFP* in transiently transformed apple leaf protoplasts. All experiments were assayed 6–12 h after bombardment. Protoplasts expressing *pCaMV35S::GFP* were used as control. Bars = 50 μm. **B**. Immunoblot assay of protein extracts from apple leaf protoplasts, cytosol, chloroplast, and mitochondria, using specific antibodies against the chloroplast protein LHCB1, the mitochondria-specific cytochrome C protein, and the cytosol-localized protein ACTIN. **C-E**. Methionine contents (**C**), ethylene production (**D**), the expression of genes in Yang’s cycle (**E**) in the WT and three *35S::MdDEP1-Myc* transgenic apple plantlets. **F**. WT and *MdDEP1* overexpression plants treated with or without 150 mg L^− 1^ AVG for 2-weeks. **G**. Chlorophyll content of plants shown in (**F**). Note: In (**C-E**) and (**G**), the data are shown as the mean ± SE, which were analyzed based on more than 9 replicates. Statistical significance was determined using Student’s *t*-test in different samples. ^*^P < 0.01; ^**^P < 0.001

Since MdDEP1 was found to play a role in chloroplast development (Fig. 1A and B), we investigated the protein localization of MdDEP1 in apple protoplasts. The pCaMV35S::MdDEP1-GFP fusion vector was constructed and introduced into protoplasts isolated from apple leaves, using the pCaMV35S::GFP as a negative control (Fig. 3A). Observations showed that the GFP signal from protoplasts expressing the pCaMV35S::MdDEP1-GFP construct was mainly enriched at regions that overlapped with chlorophyll auto-fluorescence and cytoplasm with little signal, whereas signals from the pCaMV35S::GFP construct was found throughout the whole cells (Fig. 3A). These results suggested that MdDEP1 is mainly localized in the chloroplasts and a little in cytoplasm.

Alternatively, chloroplasts, mitochondrial and cytosolic fractions were isolated from apple plants and subjected to immunoblot analysis with anti-MdDEP1 antibodies (Fig. 3B). MdDEP1 was detected mainly in the chloroplasts with a low level in the cytosolic fraction, and not in the mitochondrial fraction (Fig. 3B). Using the same protein samples, LHCB1, ACTIN and Cytochrome C were found as expected in the chloroplasts, cytosolic and mitochondrial fractions, respectively, and served as the fractionation controls (Fig. 3B). These data further confirmed that MdDEP1 is mainly located in the chloroplasts and cytoplasm.

Since DEP1 was originally discovered to function in the Yang’s cycle (Pommerrenig et al. 2011), we tested the concentrations of its key component, methionine, and the phytohormone ethylene derived from it in the *MdDEP1*-overexpression plantlets. The results showed that all three *MdDEP1*-overexpressing plantlets accumulated more methionine (Fig. 3C) and ethylene (Fig. 3D) compared to the WT. In addition, the expression of a few key genes in the Yang’s cycle were also altered by the overexpression of *MdDEP1* (Fig. 3E). The biosynthesis of ethylene was blocked by the competitive inhibitor of PLP-dependent enzymes AVG. Thus, to verify whether MdDEP1 regulation of leaf senescence depends on ethylene, WT and *MdDEP1-OVXs* apple plantlets were treated with 150 mg L^− 1^ AVG for 2 weeks, and water treatment was used as control. The results revealed that leaf senescence causes chlorophyll degradation, but anthocyanin accumulation caused by the plant’s perception of stress ultimately a little affects the visual effects of phenotypes (Fig. 3F). Both the enhanced leaf senescence and increased chlorophyll degradation phenotypes remained similar to that without the presence of AVG (Fig. 3 G). This suggested that, in addition to participating to the Yang’s cycle, MdDEP1 also played a role in promoting leaf senescence in an ethylene-independent manner.

### MdDEP1 interacts with MdY3IP1 to induce its de-phosphorylation and degradation

**Fig. 4:**
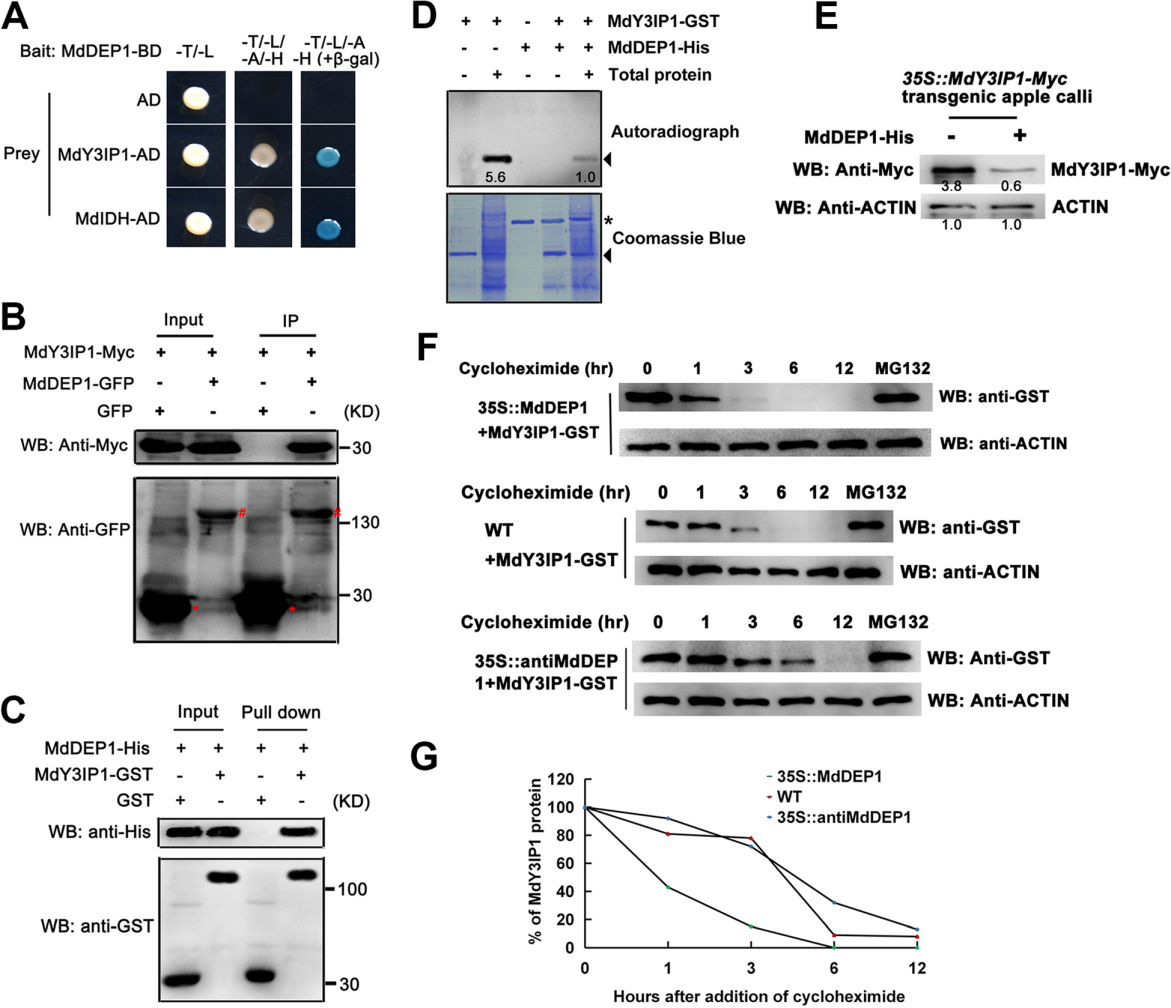
MdDEP1 interacts with, dephosphorylates and destabilizes MdY3IP1. **A**. MdDEP1 interacts with MdY3IP1 in Y2H assays. Interaction was shown by the ability of yeast cells to grow on minimal medium -Leu/−Trp/−His/−Ade with or without β-galactosidase. **B**. Co-IP assays of MdDEP1 and MdY3IP1 in apple leaf protoplasts expressing the 35S::MdDEP1-GFP and the 35S:: MdY3IP1-Myc fusion proteins. The MdDEP1-GFP proteins were immunoprecipitated with an anti-GFP antibody and immunoblotted with an anti-Myc antibody. Note: Red Asterisk and pound sign represent GFP and MdDEP1-GFP proteins, respectively. **C**. In vitro pull-down assays using antibodies against the GST tag. Both the MdDEP1-His and the MdY3IP1-GST are expressed and purified from *E. coli*. **D**. MdDEP1 dephosphorylates MdY3IP1 protein in vitro. The phosphatase assay was initiated by adding radiolabeled ATP to the mixtures. Purified proteins are from *E. coli* expression. Total protein was extracted from apple leaves. MdDEP1-His is indicated by the black asterisk. MdY3IP1-GST is indicated by black triangles. Note: Phosphorylated protein bands were quantified by scanning densitometry using a Hewlett Packard scanjet scanner and Scanplot software. **E**. *E. coli* expressed MdDEP1 facilitates the degradation of the MdY3IP1-Myc proteins expressed in the apple leaf protoplasts. Total proteins were extracted from *35S::MdY3IP1-Myc* transiently transformed apple leaf protoplasts, and incubated with *E. coli* expressed MdDEP1-His proteins or buffer control. Actin is used as a control. Note: Protein bands were quantified by scanning densitometry using a Hewlett Packard scanjet scanner and Scanplot software. **F**. Degradation assays of recombinant MdY3IP1-GST proteins in the presence of protein extracts from the WT apple leaf protoplasts or the ones expressing *35S::MdDEP1* or *35S::antiMdDEP1*. Samples were incubated in the degradation buffer with or without proteasome inhibitor (50 mM MG132). MdY3IP1-GST levels were visualized by immunoblotting using the anti-GST antibody. ACTIN was used as control. **G**. The degradation curve of MdY3IP1-GST proteins as indicated in (**F**). Quantification of the MdY3IP1-GST proteins using ImageJ software

To find more about the functions of MdDEP1, yeast two-hybrid (Y2H) screen was performed on an apple cDNA library using MdDEP1 as bait. As a result, a nucleus-encoded thylakoid protein MdY3IP1 (Yu et al. 2018) was identified (Table S1). Subsequently, the full-length cDNA of MdY3IP1 was cloned for further Y2H analysis, which showed that MdDEP1 interacted with the MdY3IP1 protein directly when MdIDH was used as a positive control (Fig. 4A). Furthermore, an in vivo co-immunoprecipitation (Co-IP) assay using the apple leaf protoplasts expressing both the *35S::MdDEP1-GFP* and the *35S::MdY3IP1-Myc* constructs was conducted. The MdY3IP1-Myc protein was immunoprecipitated together with the MdDEP1-GFP protein but not the GFP control when antibodies against the GFP were used, suggesting that MdDEP1 interacts with MdY3IP1 in vivo (Fig. 4B). In addition, an in vitro GST pull-down assay was carried out using MdY3IP1-GST and MdDEP1-His proteins that were expressed and purified from *E. coli* BL21. The results showed that the GST-tagged MdY3IP1, but not the GST alone, interacted with the His-tagged MdDEP1 protein (Fig. 4C).

Since DEP1 possesses the phosphatase activity (Pommerrenig et al. 2011), and MdDEP1 interacted with MdY3IP1 directly, it is reasonable to hypothesize that the MdDEP1 protein phosphatase mediates the dephosphorylation of MdY3IP1 protein in apple cells. In-gel assays were performed using the MdY3IP1-GST and the MdDEP1-His fusion proteins expressed and purified from *E. coli*, and the total proteins extracted from apple leaves (Fig. 4D). When incubated with leaf total protein extract, the MdY3IP1-GST proteins were clearly phosphorylated (Fig. 4D, 2^nd^ lane). However, when the MdDEP1-His proteins were present, the phosphorylation of MdY3IP1-GST protein was greatly reduced (Fig. 4D, 5^th^ lane). Given the fact that DEP1 is a phosphatase, it is very likely the phosphorylated MdY3IP1 proteins were dephosphorylated by the recombinant MdDEP1 proteins, indicating that MdY3IP1 is a direct substrate of the phosphatase activity of MdDEP1.

Interestingly, we also observed that the presence of MdDEP1 seemed to accelerate the degradation of the MdY3IP1 proteins. When *E. coli* expressed and purified MdDEP1-His proteins were added into the total proteins extracted from the apple leaf protoplasts transiently expressing the *35S::MdY3IP1-Myc* construct, the detected MdY31IP1-Myc protein was much less compared to without the presence of the MdDEP1-His protein (Fig. 4E). Additionally, to confirm this observation, a cell-free degradation assay was developed using the MdY3IP1-GST fusion proteins expressed and purified from *E. coli*, and protein samples extracted from the WT apple leaf protoplasts or protoplasts expressing the *35S::MdDEP1* or the TRV-MdDEP1 construct. The results showed that the MdY3IP1-GST proteins were more rapidly degraded in the protein extracts of the *35S::MdDEP1* apple leaf protoplasts than in those of the WT (Fig. 4 F and G). On the other hand, the MdY3IP1 proteins seemed to be more stable in the protein extracts of TRV-MdDEP1 apple leaf protoplasts compared to the WT (Fig. 4F and G). Overall, these results suggested that MdDEP1 promotes the degradation of the MdY3IP1 protein, and likely it is through protein dephosphorylation.

### MdDEP1 mainly modifies the protein abundances of the PSI subunits to suppress the photosynthesis

**Fig. 5:**
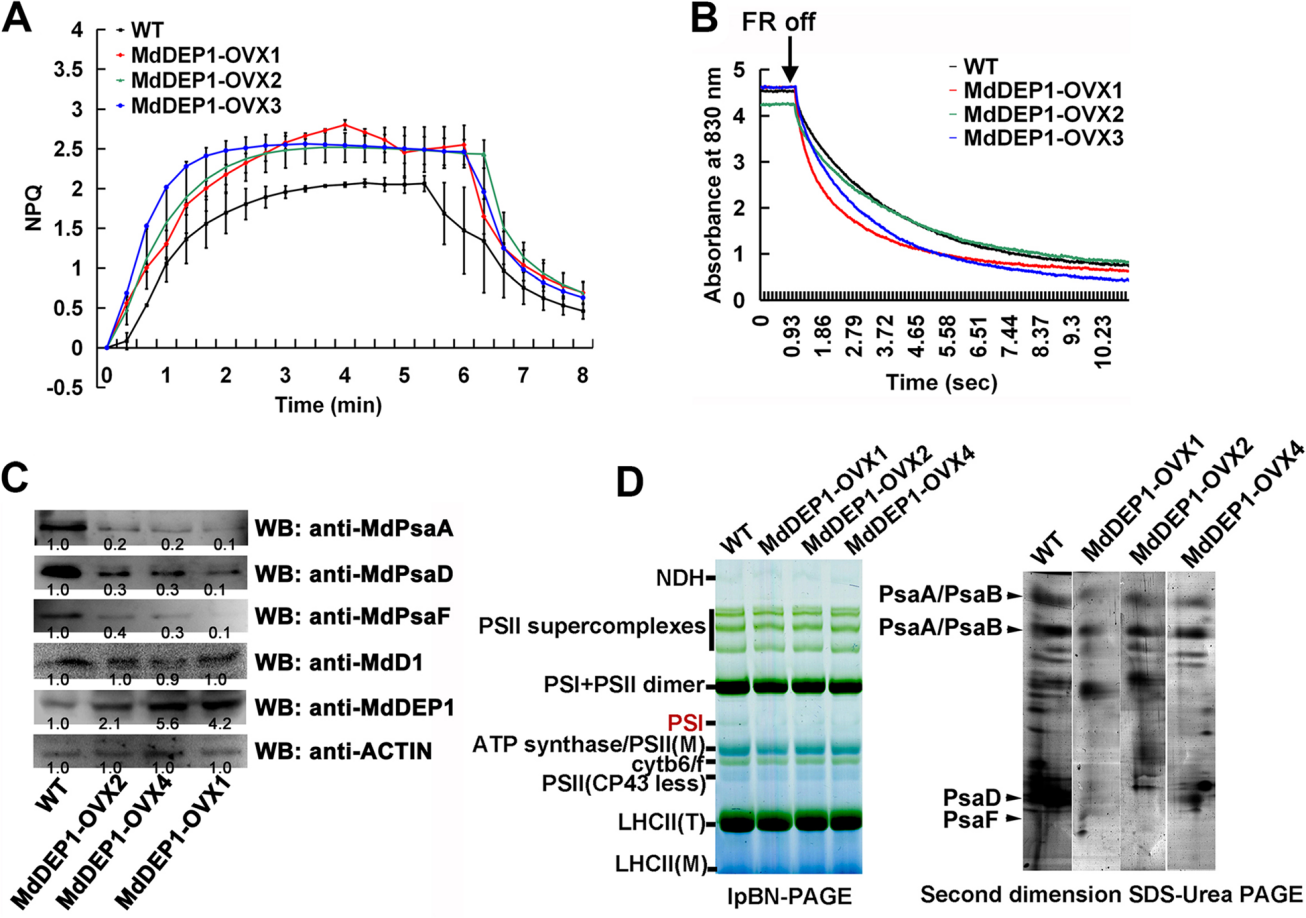
MdDEP1 attenuates PSI activity in apple leaf cells. **A**. Induction and relaxation of NPQ monitored during dark-to-light transition (120 μmol photons m^− 2^ s^− 1^). Curves represent an average of six independent measurements. **B**. Re-reduction of P_700_^+^ in darkness. P_700_ was oxidized by illumination of the leaf with far red (FR) light for 30 s and after termination of FR illumination, P_700_^+^ re-reduction was monitored in darkness. Curves, representing an average of six independent measurements, are normalized to the same amplitude for direct comparison of the kinetics. **C**. Immunoblot assays of PS I core protein subunits PsaA, PsaD, and PsaF, and PS II reaction center protein D1. An anti-MdDEP1 antibody was used to detect MdDEP1 and an anti-ACTIN antibody was used as a control. Note: Protein bands were quantified by scanning densitometry using a Hewlett Packard scanjet scanner and Scanplot software. **D**. Accumulation of PSI complexes in the WT and three *MdDEP1* transgenic apple plantlets. Thylakoids were isolated at the end of the dark period, solubilized with digitonin and protein complexes were separated by large pore blue native analysis. Gels were loaded on Chl basis. PSI complexes were identified by second dimension (BN-PAGE). A representative example from three independent biological replications is shown

To further examine the function of MdDEP1 in the photosystem, the efficiency of photosynthetic electron transport and the functional status of the photosynthetic apparatus were investigated in the *MdDEP1*-overexpressing and the WT apple plantlets. Under moderate light conditions, dramatic reductions of the PM (fraction of oxidizable PSI), the ΦI (Effective PSI quantum yield), and the ΦII (Effective PSI quantum yield) were observed in the *MdDEP1*-overexpression plantlets compared to the WT (Table S2). A slight change in PSII was found in the *MdDEP1* overexpression plantlets compared to the WT (Table S2). At the same time, there was a slight increase of the Ф_NPQ_ (Non-photochemical energy dissipation) in the *MdDEP1* transgenic plantlets, especially in the *MdDEP1-OVX1* line (Fig. 5A). Furthermore, the re-reduction of P_700_ in darkness, after termination of far red light, was noted to be faster in all three *MdDEP1*-overexpressing plantlets than that in the WT (Fig. 5B). This observation implied that a higher capacity of the *MdDEP1*-overexpressing apple plantlets for PSI cyclic electron transfer (CET) followed by protonation of the lumen, which is a prerequisite for triggering of NPQ and a photosynthetic control of the Cyt b6f complex.

The protein abundance of the photosystem core complex subunits was also examined. Immunoblotting assays showed that there was no statistically significant difference in the amount of the PSII reaction center protein D1 between *MdDEP1*-expressing plantlets and the WT control (Fig. 5C). In contrast, the PSI core complex subunits PsaA, PsaD, and PsaF all accumulated much less in those three *MdDEP1*-overexpressing plantlets than those of the WT control (Fig. 5C). Moreover, a large pore blue native polyacrylamide gel electrophoresis (lpBN-PAGE) was performed to examine the amount of low molecular mass PSI complexes, which might represent either degradation products or biogenesis intermediates. First and second dimension (BN-PAGE) revealed lower accumulations of both PSI core subcomplex and the PSI subunits including PsaA, PsaD and PsaF in the *MdDEP1*-overexpressing thylakoids compared to the WT (Fig. 5D). These observations demonstrated that the overexpression of *MdDEP1* may have mainly inhibited the photosynthesis function through decreasing the protein abundance of the PSI subunits.

### MdDEP1 stimulates ROS production

**Fig. 6:**
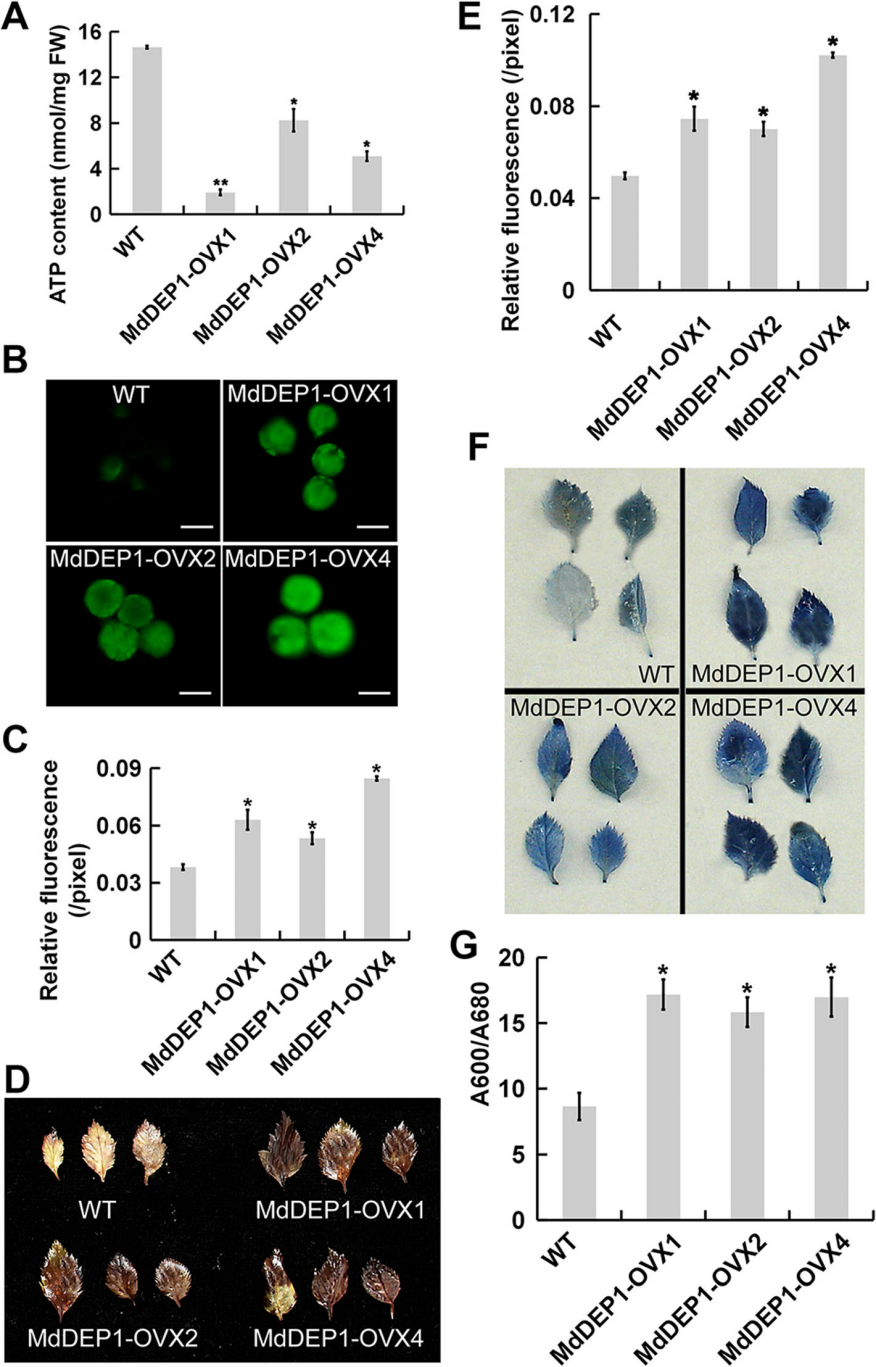
MdDEP1 triggers ROS overproduction in apple leaf cells. **A**. ATPase content in the WT and *MdDEP1* transgenic apple plantlets. **B**. Protoplasts isolated from WT and *MdDEP1*-overexpressing apple leaves were incubated in C-H_2_DCFDA for 90 s. **C**. The fluorescence signals of protoplasts in (**B**) were quantified by pixel intensity. Data represent means ± SD of 12 to 15 individual protoplasts. **D**. DAB staining for H_2_O_2_ in the leaves of the WT and *MdDEP1* transgenic apple plantlets. **E**. DAB staining intensity as determined with imageJ software. **F**. NBT staining for superoxide in the leaves of the WT and *MdDEP1* transgenic apple plantlets. **G**. NBT staining intensity as determined with imageJ software. Note: In (**A**), (**C**), (**E**), and (**G**), the data are shown as the mean ± SE, which were analyzed based on more than 9 replicates. Statistical significance was determined using Student’s *t*-test in different samples. ^*^P < 0.01; ^**^P < 0.001

In chloroplasts, PSI photochemical reactions to generate a ΔpH gradient with the Cyt b6/f complex, which could drive ATP synthesis without producing NADPH (Shikanai 2007). Since the impairment of PSI function usually leads to the reduction of ATP production and increased ROS accumulation in cells (Vass, 2012), we determined both the ATP contents and the ROS accumulations in the WT and *MdDEP1*-overexpressing plantlets. Compared to the WT, the ATP contents were significantly reduced in the *MdDEP1*-overexpressing plantlets (Fig. 6A). Subsequently, the ROS accumulation was visualized by the carboxylated 2′,7′-dichlorodihydrofluorescein diacetate [C-H_2_DCFDA] staining of protoplasts isolated from the leaves. It seemed more ROS was produced in the protoplasts isolated from the *MdDEP1*-overexpressing leaves compared to the WT controls (Fig. 6B and C). Additionally, C-H_2_DCFDA staining showed that the roots of *MdDEP1*-expressing *Arabidopsis* also accumulated more ROS than those of the WT controls (Fig. S3). Similarly, leaf staining by DAB (3,3′-diaminobenzidine) and NBT to visualize ROS accumulation both showed that the *MdDEP1*-overexpressing plantlets generated more ROS compared to the WT controls (Fig. 6D-G). Collectively, these results suggest that MdDEP1 attenuates PSI functions, and its overexpression triggers overproduction of ROS in apple cells.

## Discussion

The Yang cycle recycles 5′-methylthioadenosine (MTA) produced during the biosynthesis of ethylene, nicotianamide, or polyamines to methionine at the expense of one ATP and of newly added amino group (Pommerrenig et al. 2011). It plays important roles in ethylene-mediated leaf senescence (Grbić and Bleecker 1995; Pommerrenig et al. 2011). The photosynthetic electron transport around PSI drives the light reactions of photosynthesis, and is essential for balancing the ATP/NADPH production ratio and for protecting both photosystems from damage caused by stromal overreduction (Yamori and Shikanai 2016). These two physiological processes play vital roles in plant growth and development, as well as defense response and signal transduction in higher plants (Pommerrenig et al. 2011; Yamori and Shikanai 2016). Although a connection between the ethylene-mediated leaf senescence and the photosynthesis has been characterized (Quirino et al. 2000; Ceusters and Van de Poel 2018), the specific mechanism underlying the relationship is not well defined. The present study shows that the Yang cycle enzyme MdDEP1 attenuates PSI functions, and triggers overproduction of reactive oxygen species (ROS) in plant cells by directly interacting with and dephosphorylating the nucleus-encoded thylakoid protein MdY3IP1. These findings demonstrate that MdDEP1 is essential for photosystem activity and ethylene-independent leaf senescence due to its enzymatic function and moonlighting role in destabilizing MdY3IP1.

DEP1 is confirmed to be a trifunctional enzyme and catalyzes the dephosphorylation and the enolization of MTRu-1-P to the DHKMP (Pommerrenig et al. 2011). Consistent with its role in the Yang cycle, apple plantlets overexpressing *MdDEP1* accumulated a significantly higher amount of methionine (Fig. 3C). Furthermore, MdDEP1 was involved in the dephosphorylation of the nucleus-encoded thylakoid protein MdY3IP1 (Fig. 4D), suggesting that MdDEP1 has a phosphatase activity. These results support a functional conservation of Yang cycle enzyme DEP1 in higher plants. Very interestingly, Zierer et al. (2016) show that *Arabidopsis* DEP1 is a cytosolic enzyme. However, we found that apple DEP1 was detected mainly in the chloroplasts with a low level in the cytosolic fraction (Fig. 3A and B). This subcellular localization difference of DEP1 between *Arabidopsis* and apple is likely owing to their sequence similarity with significantly large differences, suggesting that there may be some differences in protein functions between woody plants and herbaceous plants (Fig. S4). Additionally, Yao et al. (2007) found that *MdDEP1* contributes to lowering the acidity of fruit in apple. Based on our findings presented here, it is possible that the overexpression of *MdDEP1* may have reduced the PSI activity and inhibited the overall photosynthesis performance, which in turn may have inhibited the accumulation of the organic acids accordingly.

The PSI is a large multiprotein complex in the thylakoid membrane, and its intricate assembly depends on auxiliary protein factors. One of the essential assembly factors for PSI is encoded by *Ycf3* in the chloroplast genome (Albus et al. 2010; Rochaix 2011a). The Ycf3 protein contributes to an efficient PSI assembly by directly interacting with the nucleus-encoded thylakoid protein Y3IP1 in tobacco and *Arabidopsis* (Albus et al. 2010). Additionally, Y3IP1 plays key roles in photosynthetic carbohydrate synthesis by modulating the PSI activity in *Arabidopsis* and apple (Albus et al. 2010; Yu et al. 2018). Y3IP1 acts at the post-translational level and is a novel assembly factor of PSI (Albus et al. 2010). So we inferred that the Yang cycle enzyme MdDEP1 triggers ROS overproduction and alters PSI activity in plant cells, by directly interacting with, as well as dephosphorylating and destablizing the nucleus-encoded thylakoid protein MdY3IP1 (Fig. 4; Fig. 6). Combined with the Yang cycle and the photosynthesis-inhibited phenotypes by methionine in plant leaves (Krapp et al. 1993; Pommerrenig et al. 2011; Ceusters and Van de Poel 2018), we have connected two important physiological processes of the Yang cycle and cyclic electron transport around PSI.

**Fig. 7:**
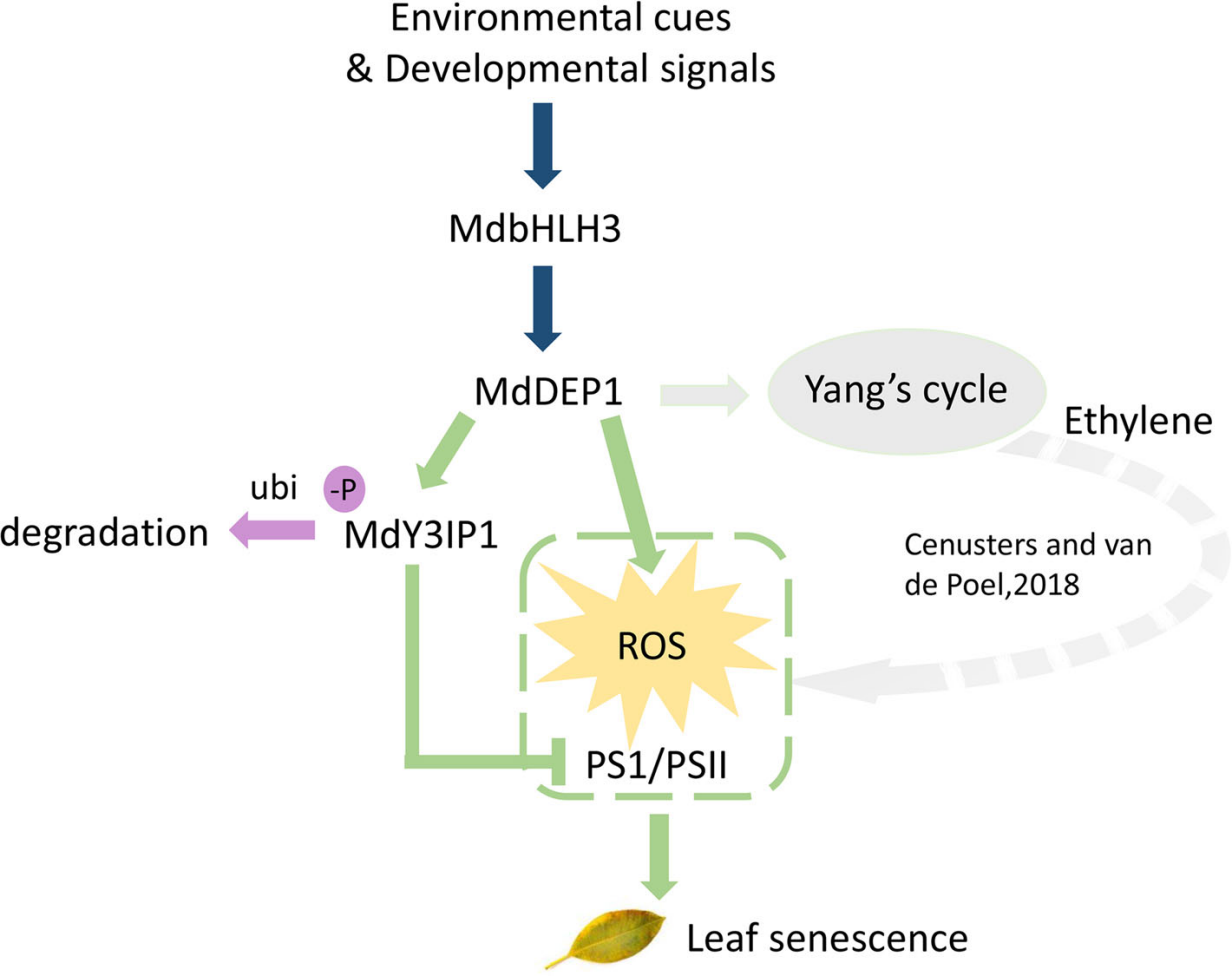
The moonlighting protein MdDEP1 functions in two different directions to eventually regulate/control leaf senescence. On one direction, MdDEP1 activates the Yang’s cycle to promote ethylene production and signaling, which will weaken the function of photosystems and promote ROS production. On the other direction, MdDEP1 promote the MdY3IP1 dephosphorylation, whose degradation also inhibits the function of photosystems and promote ROS production. The weakened PSI/PSII functions and increased ROS production will lead to leaf senescence

Taken together, combine our previous study (Hu et al., 2020), the data support a thesis in which the moonlighting protein MdDEP1 functions in two different directions to eventually regulate/control leaf senescence (Fig. 7). MdbHLH3 could transcription activates Yang cycle phosphatase *MdDEP1*. On one direction, MdDEP1 activates the Yang’s cycle to promote ethylene production and signaling, which will weaken the function of photosystems (Ceusters and Van de Poel 2018; Table S3) and promote ROS production. On the other hand, MdDEP1 dephosphorylates and destabilizes the nucleus-encoded thylakoid protein MdY3IP1, whereas an unknown protein kinase phosphorylates and stabilizes MdY3IP1 to modulate PSI functions and ROS homeostasis, as well as ROS-triggered phenotype and leaf senescence, in response to environmental cues and developmental signals. Negative regulation of MdDEP1 is more powerful than the positive regulation of protein kinase, resulting in the reduction of the MdY3IP1 proteins and the PSI activity, as well as an increase of the ROS accumulation and accelerated leaf senescence. Conversely, positive regulation of the unknown protein kinase is more powerful than the negative regulation of MdDEP1, resulting in an enhancement of MdY3IP1 proteins and the PSI activity, as well as a reduction of ROS accumulation and delayed leaf senescence. Therefore, our findings suggest a regulatory mechanism in which MdDEP1 and the unknown protein kinase fine-tune the PSI activity and ROS homeostasis by modulating the stability of the MdY3IP1 proteins in response to environmental cues and developmental signals in apples or other plant species.

The ethylene-mediated leaf senescence and photosynthesis are two physiological processes that play critical roles in plant growth and development, as well as defense response in higher plants (Pommerrenig et al. 2011; Yamori and Shikanai 2016; Ceusters and Van de Poel 2018). Elucidating the mechanisms underlying the relationship of these two physiological processes is a key step to understanding a series of biological phenotypes (Giovannoni et al. 2017). Additionally, these characteristics are major targets of breeding programs on many crop plants. Our findings shed light on the current understanding of the roles of the moonlighting enzyme MdDEP1 in modulation of photosystems and leaf senescence. Overall, these findings provide a novel molecular basis for these important processes in plants. These may also be useful in developing novel biotechnological strategies and tools applicable in phytoremediation.

## Methods

### Plant materials and growth conditions

In vitro tissue culture plantlets of apple cultivar ‘Gala’ were maintained at room temperature under long-day conditions (16 h/8 h; light/dark) on Murashige and Skoog (MS) medium plus 0.2 mg L^− 1^ indole-3-acetic acid (IAA) and 0.8 mg L^− 1^ 6-benzylaminopurine (6-BA), and subcultured once a month. For rooting treatment, 3-week-old tissue plantlet cultures were transferred to the root-inducing MS medium supplemented with 0.1 mg L^− 1^ IAA. The overexpression apple leaves used for determination were obtained from the stage, which was defined according to the characteristics of leaf age (T3: 45 day) in reference to the second leaf from the bottom of the apple shoot.

### RNA extraction, RT-PCR and qRT-PCR assays

RNA extraction, RT-PCR and qRT-PCR assays were performed as described in Hu et al. (2016a). See Table S4 for all primer sequences.

### Plasmid construction and genetic transformation

Sense full-length sequences of *MdDEP1* were amplified to construct sense overexpression vectors. The resulting PCR products were inserted into the pCXSN::Myc vector under the control of the *35S* promoter. This construct was genetically transformed into tissue cultures of apple cultivar ‘Gala’ using *Agrobacterium* (LBA4404)-mediated transformation as described previously (Hu et al. 2016a).

### Construction of the transient expression vectors in apple leaves

To construct the antisense expression viral vectors, the cDNA fragments of *MdDEP1* was amplified with RT-PCR assay. The PCR products were cloned into the tobacco rattle virus (TRV) vector in the antisense orientation under the control of the dual *35S* promoter. The resultant vector was named TRV-MdDEP1. The TRV-MdDEP1 construct was transformed into the *Agrobacterium tumefaciens* strain GV3101 for inoculation. The functional leaves of tissue culture plantlets growing one month were selected for infection. The transfection of apple leaves was performed as described in Hu et al. (2016a). Phenotypic observation and index detection were performed after 7 days of infection.

### RNA-Seq analysis

The total RNAs were extracted from the leaves of the WT and *MdDEP1-*overexpressing or viral vector-based *MdDEP1*-silencing apple plantlets. Subsequently, the RNAs for these leaf samples were used to construct libraries for high-throughput parallel sequencing using an Illumina genome analyzer II. A rigorous algorithm was used to identify the differentially expressed genes in these samples. The false discovery rate (FDR) was set at 1% to determine the threshold of the *P*-value in multiple tests and analyses by manipulating the FDR value (Audic and Claverie 1997). *P* < 0.001 and the absolute value of log2Ratio > 1.5 were used as the threshold to determine the significance of the gene expression differences according to Audic and Claverie (1997). A gene ontology (GO) analysis was used to predict gene function and calculate the functional category distribution frequency.

### Protein extraction and immunoblotting assays

Protein extraction and immunoblotting assays were conducted as described by Hu et al. (2016b). The monoclonal antibodies of anti-MdDEP1, anti-ACTIN, anti-Myc, anti-GFP, as well as anti-His and anti-GST were prepared by the Abmart Company (Shanghai, China).

### Y2H, co-IP and GST pull-down assays

Yeast two-hybrid assays were performed using the Matchmaker GAL4-based two-hybrid system (Clontech, Palo Alto, CA, USA). Full-length cDNA of MdDEP1 were inserted into the pGBT9 vector. The associated yeast two-hybrid vectors of MdY3IP1 and MdIDH, which were inserted into vector pGAD424, are detailed in Hu et al. (2016b). All of the constructs were transformed into yeast strain AH109 using a lithium acetate method. Yeast cells were cultured on minimal medium -Leu/−Trp according to the manufacturer’s instructions. Transformed colonies were plated onto minimal medium -Leu/−Trp/−His/−Ade with or without β-galactosidase to test for possible interactions. The *35S::GFP* and *35S::MdDEP1-GFP* transgenic apple calli were treated with 50 μM MG132 for 16 h to stabilize the MdDEP1-GFP and MdY3IP1-Myc proteins. The Co-IP was carried out as described by Hu et al. (2016b). The eluted samples were immunoblotted using anti-GFP and anti-Myc antibodies. For the GST pull-down assays, full-length cDNA of *MdY3IP1* were inserted into the pGEX-4 T-1 vector, whereas that of *MdDEP1* was inserted into pET-32a-c. All of the recombinant proteins were used to perform GST pull-down assays as described by Hu et al. (2016b).

### Cell-free degradation

Cells (*E. coli*, *BL21*) were induced by 0.1 mM IPTG and allowed to grow for 12 h at 16 °C. MdY3IP1-GST protein was eluted from glutathione–agarose beads. The total proteins of the transgenic apple leaf protoplasts were subsequently extracted in degradation buffer containing 25 mM Tris-HCl (pH 7.5), 10 mM NaCl, 10 mM MgCl_2_, 4 mM PMSF, 5 mM DTT and 10 mM ATP as previously described by Zhao et al. (2016). The supernatant was collected, and the protein concentration was determined by the using the Bradford assay reagent (Bio-Rad, Hercules, CA, USA). Each reaction mix contained 100 ng of MdY3IP1-GST protein and 500 μg of total protein from WT, *35S::MdDEP1* and TRV-MdDEP1 apple leaf protoplasts. The reaction mixes were incubated at 22 °C, and were stopped by the addition of SDS-PAGE sample buffer and boiled for 10 min. The results were quantified using Quantity One 1-D Analysis Software (Bio-Rad, Hercules, CA, USA).

### Determination of methionine content

Freeze-dried whole plant samples (50 mg) were used for the determination of methionine content as described previously (Ishimoto et al. 2010). Free methionine was extracted with 240 μL of 3% sulfosalicylic acid for 60 min followed by centrifugation at 12,000×g for 10 min at room temperature. The obtained pellet was additionally extracted two times with the same amount of extraction solution at shaking for 60 min and were combined together and the resultant suspension were further processed for amino analysis. Samples of all the treatment were hydrolyzed in 5 ml of 6 M HCl under vacuum in an ampulla tube for 24 h at 110 °C. The suspension was then filtered and evaporated under vacuum. The solid residue was dissolved in 2 ml of deionized water and evaporated twice again. The final residue was dissolved in 10 ml of 0.01 M HCl and then filtered with a 0.22-μm filter membrane and subjected to an automatic amino acid analyzer (L-8900 Hitachi, Japan). An amino acid standard mixture solution (type H) for automatic amino acid analysis was purchased from Wako Pure Chemical Industries Ltd. (Japan) and used for quantification of endogenous methionine levels.

### AVG treatment of apple plantlets

Two-months-old wild-type and three *35S::MdDEP1-Myc* transgenic apple plantlets were selected for the experiment. The experiment was divided into control group and AVG treatment group. 150 mg L^− 1^ AVG was evenly sprayed on the leaves of apple plantlets, while water was used as the control. The treated plantlets were placed under long-day condition for 2 weeks to observe phenotypes and test indexes.

### Protoplast isolation

The tender leaves were collected from apple plants grown under optimal light condition (ca. 150 μE m^− 2^ s^− 1^), rinsed with excessive sterile water, and briefly dried. The lower epidermis were tore off with a sharp-pointed tweezer, and then quickly transferred these leaf lower epidermis to 10 ml of enzymolysis solution of cell wall containing [1.5% cellulase ‘Onozuka’ R10 (Yakult, Tokyo, Japan), 0.4% macerozyme ‘Onozuka’ R10 (Yakult, Tokyo, Japan), 0.05% pectolase Y-23 (Yakult, Tokyo, Japan), 0.4 M mannitol, 10 mM CaCl_2_, 20 mM KCl, 20 mM MES and 2% sucrose, pH 5.8], and were placed at room temperature in the dark for 12 h. Subsequently, the enzyme solution including leaf lower epidermis were gently shaken (30 rpm on a platform shaker) in light for 30 min so that the protoplasts were completely released into the solution. Add the same volume of prechilled modified W5 solution (154 mM NaCl, 125 mM CaCl_2_, 5 mM KCl, and 2 mM MES, pH 5.8), and then filtered with 75 μm nylon fabric in 50 ml centrifuge tube. The protoplasts were centrifuged at 500 rpm for 10 min, and then removed the supernatant. The protoplasts at the bottom pipe were repeatedly washed twice with 10–15 mL of prechilled modified W5 solution, and incubated on ice for 30 min. During the incubation period, the protoplasts were counted using a hemocytometer under a light microscope. The protoplasts were then centrifuged and resuspended in prechilled modified MMg solution (0.4 M mannitol, 15 mM MgCl_2_, and 4 mM MES, pH 5.8) to a final concentration of 1 to 5 × 10^4^ cells/mL.

### Protoplast transfection assays

Protoplasts were transfected by a PEG-mediated method as described by Hu et al. (2019) with a minor modification. Approximately 5 × 10^4^ protoplasts in 1 mL of MMg solution were mixed with about 30 μg of plasmid DNA in 10 ml centrifuge tube at room temperature. 1 mL of a freshly-prepared 40% PEG4000 (w/v) solution including 0.1 M CaCl_2_ and 0.2 M mannitol was added, and the mixture was incubated for 5 min at room temperature. After incubation, 6 mL of W5 solution was added and mixed slowly. The mixture was then centrifuged at 400 rpm for 5 min, and removed the supernatant. The transfected protoplasts were washed twice with W5 solution. Subsequently, the protoplasts were gently resuspended in 1 mL of WI solution (0.5 M mannitol, 20 mM KCl, 4 mM MES, pH 5.8), and were transferred in 6-well plates. These protoplasts were expressed in dim light for no more than 9 h.

### Subcelluar localization analysis

Protoplasts isolated from cells of apple leaves were prepared and transformed as described by Hu et al. (2016a). Fluorescence derived from transformed apple protoplasts was detected with a confocal laser scanning microscope (Zeiss LSM 510 META, Jena, Germany) (Zhang et al. 2021). A total of 20–30 apple protoplasts were imaged for this experiment.

### Confocal laser microscopic and electron microscopic examination

Transmission electron microscopic studies of apple tissues followed the standard method described by Spurr (1969). The samples were observed through a JEOL 100CX transmission electron microscope (Jeol, Peabody, MA, USA).

### DAB, NBT, and H_2_DCFDA staining for ROS accumulation

In situ ROS accumulation in apple leaves was examined via histochemical staining with DAB and NBT, respectively. ROS content were quantified according to the method of Zhao et al. (2016).

ROS accumulation in protoplasts was determined by staining with the fluorescent dye C-H_2_DCFDA (Sigma-Aldrich). For C-H_2_DCFDA staining, protoplasts were treated with 20 mM C-H_2_DCFDA (Invitrogen) for 30 min and subsequently imaged using an inverted laser scanning confocal microscope (LSM780; Zeiss) with an excitation at 488 nm. The imageJ was used for quantifying ROS intensity.

### Statistical analysis

All samples were in triplicates, and all data represented as the mean ± standard deviation unless labeled separately. Significances were determined using Student’s *t* test (*p* ≤ 0.01 was considered to be significant, *p* ≤ 0.001 represented a very significant difference, while n.s. was no significance).

### Supplementary Information

**Additional file 1: Fig. S1.** *MdDEP1* is highly associated with leaf senescence in apple.

**Additional file 2: Fig. S2.** qRT-PCR to confirm RNA-seq data. A. Expression level of genes involved in senescence. B. Expression level of genes involved in photosystem I (PS I) genes. C. Expression level of genes involved in photosystem II (PS II) genes.

**Additional file 3: Fig. S3.** ROS accumulation in the roots of wild-type (col) and three *35S::MdDEP1-GFP* transgenic *Arabidopsis* plants.

**Additional file 4: Fig. S4.** Phylogenetic tree of DEP1 proteins isolated from different plant species.

**Additional file 5: Table S1.** Identification of MdDEP1-interacting proteins in co-immunoprecipitation using an LC/MS assay.

**Additional file 6: Table S2.** Functional characteristics of the thylakoid membrane of WT and *MdDEP1* transgenic apple plants.

**Additional file 7: Table S3.** Functional characteristics of thylakoid membrane of apple plants treated with 0.2 mM ethephon and control.

**Additional file 8: Table S4.** The primers used for RT-PCR and qRT-PCR in this study.

**Additional file 9: Appendix S1.** Annotation of all unigenes in the WT and *MdDEP1-*overexpressing apple plantlets.

**Additional file 10: Appendix S2.** Annotation of all unigenes in the WT and the viral vector-based *MdDEP1*-silencing apple plantlets.

## Data Availability

Not applicable.

## Abbreviations

MdDEP1: Dehydratase-enolase-phosphatase-complex1
PSI: Photosystem I
ROS: Reactive oxygen species
PSII: Photosystem II
Cyt: Cytochrome
RC: Reaction center
Chls: Chlorophylls
PGR5: PROTON GRADIENT REGULATION 5
LHCI: Light-harvesting chlorophyll protein complex I
Ycf3: Hypothetical chloroplast reading frame number 3
Ycf4: Hypothetical chloroplast reading frame number 4
SAGs: Senescence-associated genes
SAM: S-adenosyl methionine
ACC: 1-aminocyclopropane-1-carboxylic acid
AVG: Aminoethoxyvinylglycine
ACO: ACC oxidase
Yang cycle: Methionine salvage pathway
MTRu-1-P: 5-methylthioribulose-1-P
DHKMP: 1,2-dihydroxy-3-keto-5-methylthiopentene
GO: Gene Ontology
AVG: Aminoethoxyvinylglycine
CO-IP: Co-immunoprecipitation
DAB: 3,3′-diaminobenzidine
MTA: 5′-methylthioadenosine
IAA: Indole-3-acetic acid
6-BA: 6-benzylaminopurine
FDR: False discovery rate
