# Supplementary material for: Yang cycle enzyme DEP1: its moonlighting functions in PSI and ROS production during leaf senescence
Source: Mol Hortic. 2022 Apr 20;2:10. doi: 10.1186/s43897-022-00031-2 (PMC10514949; doi:10.1186/s43897-022-00031-2)
Supplement: Supplementary file 1 — Additional file 1: Fig. S1. MdDEP1 is highly associated with leaf senescence in apple. [file 43897_2022_31_MOESM1_ESM.pdf]

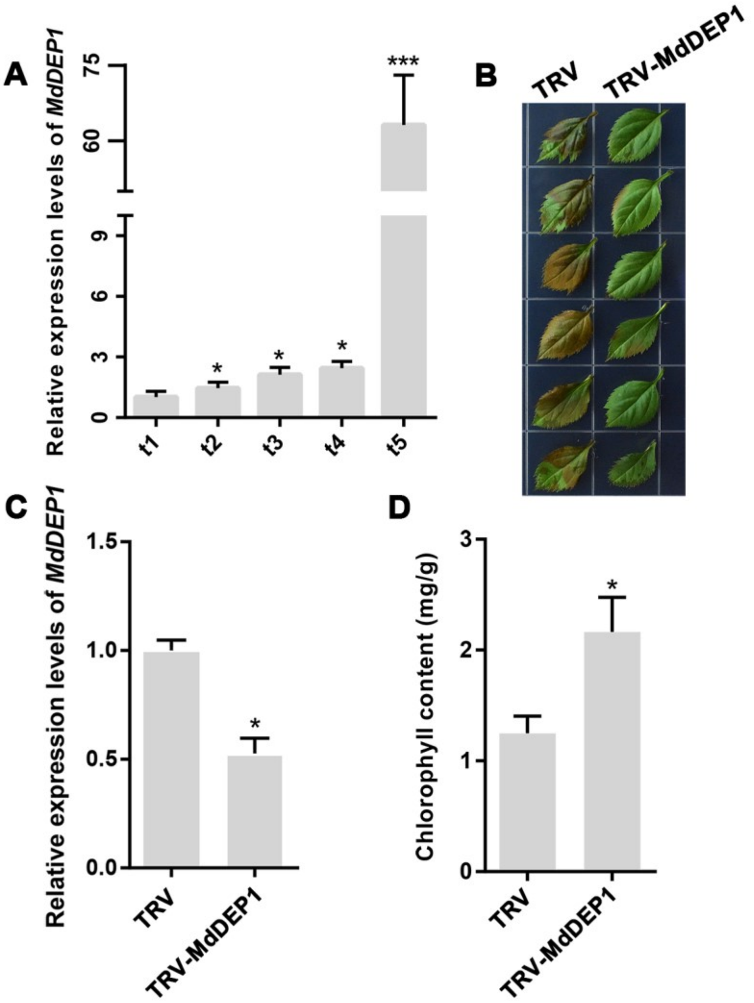

Fig. S1 *MdDEP1* responds to senescence. A. The relative expression level of MdDEP1 in leaves at different developmental stages (The whole apple seedling two months after rooting with 17 leaves to experiment. Leaves are counted from the youngest leaf at the top to the root and t1-t5 corresponds to the 1st, 3rd, 6th, 10th and 15th leaves respectively). B. TRV-MdDEP1 was transiently transformed into leaves for 7 days to observe the senescence response, and TRV was injected as control. C. Expression of MdDEP1 in transient transformation leaves. D. Chlorophyll content in TRV-MdDEP1 leaves.
