# Supplementary material for: Yang cycle enzyme DEP1: its moonlighting functions in PSI and ROS production during leaf senescence
Source: Mol Hortic. 2022 Apr 20;2:10. doi: 10.1186/s43897-022-00031-2 (PMC10514949; doi:10.1186/s43897-022-00031-2)
Supplement: Supplementary file 2 — Additional file 2: Fig. S2. qRT-PCR to confirm RNA-seq data. A. Expression level of genes involved in senescence. B. Expression level of genes involved in photosystem I (PS I) genes. C. Expression level of genes involved in photosystem II (PS II) genes. [file 43897_2022_31_MOESM2_ESM.pdf]

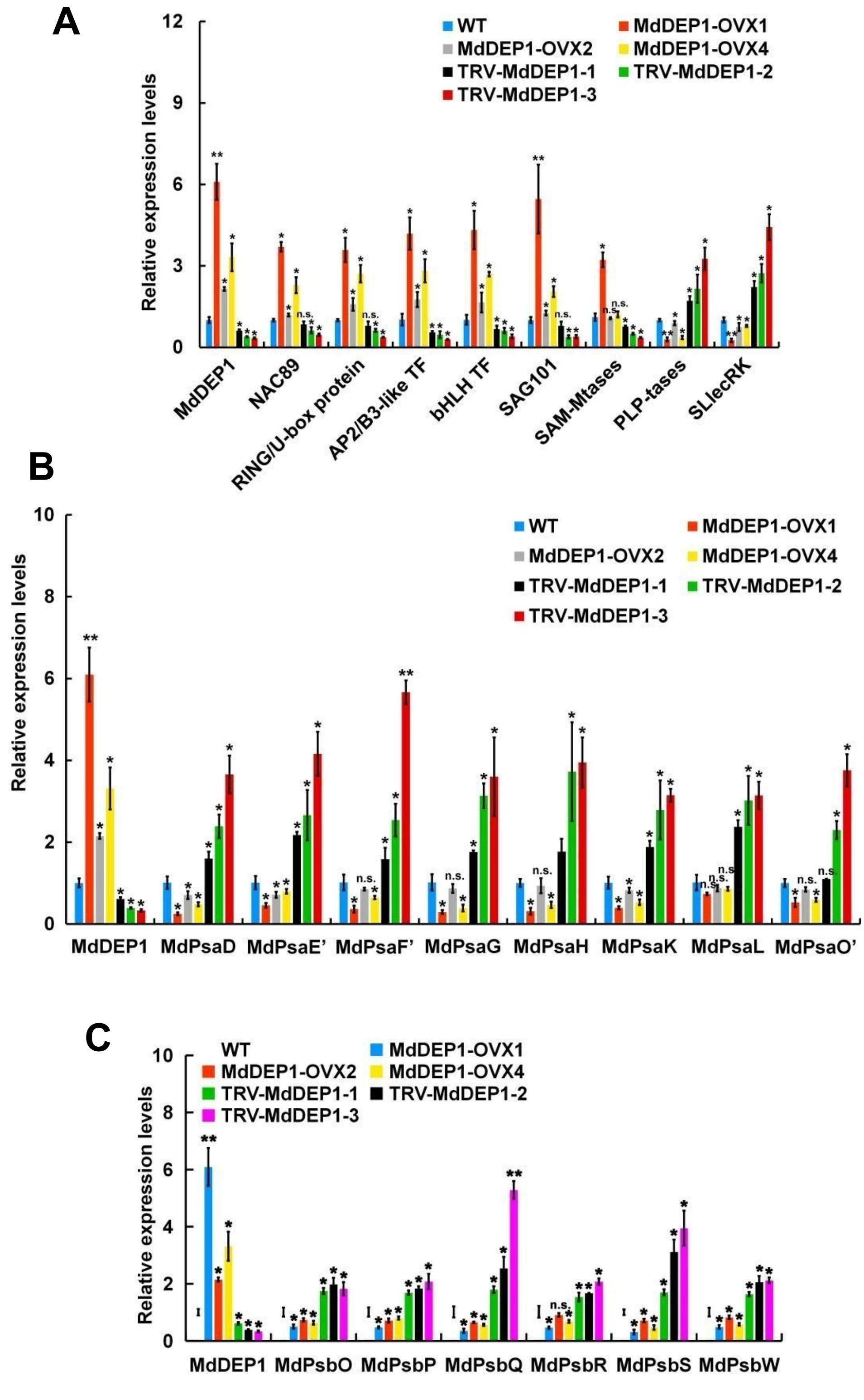

Fig. S2 qRT-PCR to confirm RNA-seq data. A. Expression level of genes involved in senescence. B. Expression level of genes involved in photosystem I (PS I) genes. C. Expression level of genes involved in photosystem II (PS II) genes.
