# Supplementary material for: Yang cycle enzyme DEP1: its moonlighting functions in PSI and ROS production during leaf senescence
Source: Mol Hortic. 2022 Apr 20;2:10. doi: 10.1186/s43897-022-00031-2 (PMC10514949; doi:10.1186/s43897-022-00031-2)
Supplement: Supplementary file 3 — Additional file 3: Fig. S3. ROS accumulation in the roots of wild-type (col) and three 35S::MdDEP1-GFP transgenic Arabidopsis plants. [file 43897_2022_31_MOESM3_ESM.pdf]

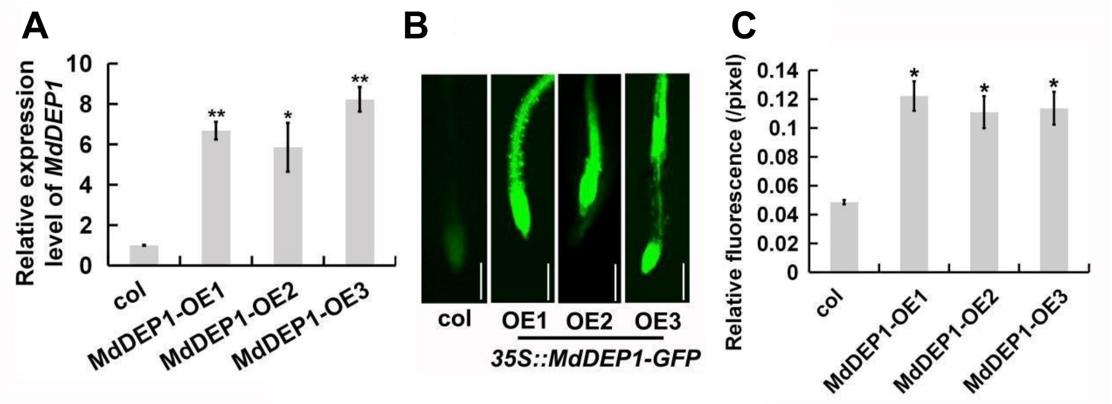

Fig. S3 ROS accumulation in the roots of wild-type (col) and three *35S::MdDEP1-GFP* transgenic *Arabidopsis* plants. A. The transcript of *MdDEP1* in the wild-type (col) and three *35S::MdDEP1-GFP* transgenic *Arabidopsis* plants. B. C-H_2DCFDA staining for ROS accumulation in the wild-type (col) and three *35S::MdDEP1-GFP* transgenic *Arabidopsis* plants. C. C-H_2DCFDA staining intensity as determined with imageJ software. Note: In (A) and (C), the data are shown as the mean ± SE, which were analyzed based on more than 9 replicates. Statistical significance was determined using Student's *t*-test in different samples. $^{*}P < 0.01$; $^{**}P < 0.001$.
