# Supplementary material for: Yang cycle enzyme DEP1: its moonlighting functions in PSI and ROS production during leaf senescence
Source: Mol Hortic. 2022 Apr 20;2:10. doi: 10.1186/s43897-022-00031-2 (PMC10514949; doi:10.1186/s43897-022-00031-2)
Supplement: Supplementary file 4 — Additional file 4: Fig. S4. Phylogenetic tree of DEP1 proteins isolated from different plant species. [file 43897_2022_31_MOESM4_ESM.pdf]

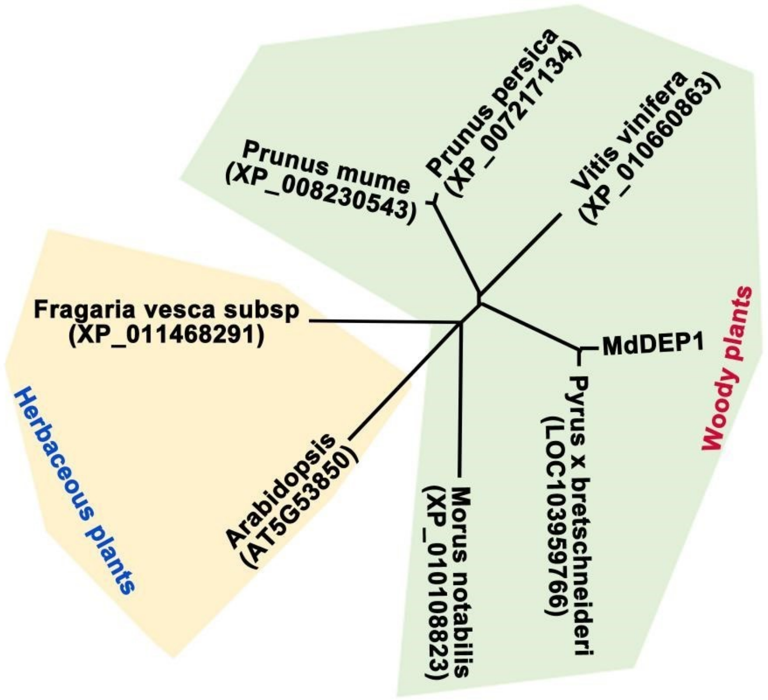

Fig. S4 Phylogenetic tree of DEP1 proteins isolated from different plant species. The tree was constructed with the MEGA 4.0 by the neighbor-joining (NJ) method with amino acid sequences of apple MdDEP1 and DEP1s from other plant species.
