# Supplementary material for: Yang cycle enzyme DEP1: its moonlighting functions in PSI and ROS production during leaf senescence
Source: Mol Hortic. 2022 Apr 20;2:10. doi: 10.1186/s43897-022-00031-2 (PMC10514949; doi:10.1186/s43897-022-00031-2)
Supplement: Supplementary file 5 — Additional file 5: Table S1. Identification of MdDEP1-interacting proteins in co-immunoprecipitation using an LC/MS assay. [file 43897_2022_31_MOESM5_ESM.pdf]

Table S1. Identification of MdDEP1-interacting proteins in co-immunoprecipitation using an LC/MS assay.

Screened gene accession NO.	Gene annotation
MDP0000033325	Arf (ADP-ribosylation factor)/ Arl (Arf-like) small GTPases
MDP0000149828	MAP Kinase, Catalytic domain of the Serine/Threonine Kinases
MDP0000184069	No apical meristem (NAM) protein
MDP0000200737	ACC oxidase, 2OG-Fe(II) oxygenase superfamily
MDP0000259742	ADP glucose pyrophosphorylase small subunit 1-like protein
MDP0000215789	Type II secretion system F domain protein
MDP0000184563	isocitrate dehydrogenase [NADP], chloroplastic-like
MDP0000797453	E3 ubiquitin-protein ligase RMA1H1-like RING finger protein
★ MDP0000930948	Encodes a chloroplast protein that induces tolerance to multiple environmental stresses and reduce photooxidative damage

Yeast two hybrid screening apple fruit yeast AD library with MdDEP1-BD.
