# Supplementary material for: Yang cycle enzyme DEP1: its moonlighting functions in PSI and ROS production during leaf senescence
Source: Mol Hortic. 2022 Apr 20;2:10. doi: 10.1186/s43897-022-00031-2 (PMC10514949; doi:10.1186/s43897-022-00031-2)
Supplement: Supplementary file 6 — Additional file 6: Table S2. Functional characteristics of the thylakoid membrane of WT and MdDEP1 transgenic apple plants. [file 43897_2022_31_MOESM6_ESM.pdf]

Table S2. Functional characteristics of the thylakoid membrane of WT and *MdDEP1* transgenic apple plants.

Photosynthetic parameter	WT	*MdDEP1-OVX1*	*MdDEP1-OVX2*	*MdDEP1-OVX4*
Fraction of oxidizable PSI, P_M	1.17±0.11	0.56±0.04*	0.85±0.01*	0.73±0.02*
Effective PSI quantum yield, Φ_I	0.42±0.03	0.29±0.04*	0.40±0.01*	0.35±0.04*
PSI donor side limitation, Φ_{ND}	0.51±0.02	0.59±0.04*	0.53±0.01*	0.56±0.02*
PSI acceptor side limitation, Φ_{NA}	0.07±0.01	0.13±0.04	0.07±0.01	0.09±0.02
Effective PSI quantum yield, Φ_{II}	0.30±0.04	0.17±0.02*	0.27±0.01*	0.26±0.05*
Yield of non-regulated non-photochemical energy loss, Φ_{NO}	0.24±0.01	0.24±0.01	0.21±0.01	0.21±0.01
Non-photochemical energy dissipation, Φ_{NPQ}	0.47±0.01	0.59±0.03*	0.52±0.01*	0.53±0.04*
Excitation pressure of PSII, 1-qP	0.51±0.07	0.34±0.01*	0.49±0.01	0.48±0.09

MdDEP1 transgenic line include *MdDEP1-OVX1*, *MdDEP1-OVX2* and *MdDEP1-OVX4*. Values were measured from plants grown under moderate light intensities (210 μmol photons m^{-2} s^{-1}).Values were measured from plants grown under moderate light intensities (210 μmol photons m^{-2} s^{-1}).The values are the means±SD, n=6-9. Statistically significant differences comparing the MdDEP1 transgenic apple plants to that of the corresponding WT are marked with asterix (*). See text for details. WT, wild-type.
