# Supplementary material for: Yang cycle enzyme DEP1: its moonlighting functions in PSI and ROS production during leaf senescence
Source: Mol Hortic. 2022 Apr 20;2:10. doi: 10.1186/s43897-022-00031-2 (PMC10514949; doi:10.1186/s43897-022-00031-2)
Supplement: Supplementary file 7 — Additional file 7: Table S3. Functional characteristics of thylakoid membrane of apple plants treated with 0.2 mM ethephon and control. [file 43897_2022_31_MOESM7_ESM.pdf]

Table S3. Functional characteristics of thylakoid membrane of apple plants treated with 0.2 mM ethephon and control.

Photosynthetic parameter	Control	0.2 mM Ethephon
Fraction of oxidizable PSI, P_M	1.16±0.02	0.60±0.01*
Effective PSI quantum yield, Φ_I	0.42±0.02	0.28±0.02*
PSI donor side limitation, Φ_{ND}	0.52±0.01	0.57±0.02*
PSI acceptor side limitation, Φ_{NA}	0.07±0.01	0.07±0.01
Effective PSI quantum yield, Φ_{II}	0.31±0.01	0.17±0.01*
Yield of non-regulated non-photochemical energy loss, Φ_{NO}	0.24±0.01	0.24±0.01
Non-photochemical energy dissipation, Φ_{NPQ}	0.47±0.02	0.60±0.02*
Excitation pressure of PSII, 1-qP	0.51±0.01	0.35±0.01*

Values were measured from plants grown under moderate light intensities (210 µmol photons $m^{-2}s^{-1}$). The values are the means±SD, n=6-9. Statistically significant differences comparing apple plants treated with 0.2 mM ethephon to that of the corresponding control (Untreated apple plants) are marked with asterix (*). See text for details.
