# Supplementary material for: Yang cycle enzyme DEP1: its moonlighting functions in PSI and ROS production during leaf senescence
Source: Mol Hortic. 2022 Apr 20;2:10. doi: 10.1186/s43897-022-00031-2 (PMC10514949; doi:10.1186/s43897-022-00031-2)
Supplement: Supplementary file 8 — Additional file 8: Table S4. The primers used for RT-PCR and qRT-PCR in this study. [file 43897_2022_31_MOESM8_ESM.pdf]

Table S4 The primers used for RT-PCR and qRT-PCR in this study.

Name	Primer sequences	Note
MdDEP1-F(+A)	AATGGCAGCGGAAGCTGAGGTTC	Full length primers of *35S::MdDEP1-*
MdDEP1-R(+A)	TATCATGCTAGCCTCTTCTTTTAG	*GFP/35S::MdDEP1-Myc*
pMdDEP1-F	GTAACATCTCACATCAACCAACG	The primers of *MdDEP1* promoter
pMdDEP1-R	TTGCCACAGTCGTATGATTCATC	
MdDEP1-F(pIR)	GTCGACGAAGGCGGACATCTGACAAC	The sense pIR virus vector of
MdDEP1-R(pIR)	TCTAGACTTATCTATGTTCTCTTCTGAGT	*35S::MdDEP1*
MdDEP1-F(TRV)	TCTAGAAAGCATGGTACTCTTTCGGTCTAC	The antisense TRV virus vector of
MdDEP1-R(TRV)	GGATCCATCGTGCGGCTCCAATTAAATGTT	*35S::MdDEP1*
MdY3IP1-F(+A)	AATGGGTTTGCAAATTCTCC	Full length primers of *35S::MdY3IP1-*
MdY3IP1-R(+A)	CTGGAGCGAATTGTAAAACA	*Myc*
MdDEP1-F(Y2H)	GGATCCTGGAAGGCGGACATCTGAC	Yeast two hybrid primers
MdDEP1-R(Y2H)	GTCGACTCACATCAGCTACCTCAGT	
MdY3IP1-F (AD-EcoRI)	GAATTCATGGGTTTGCAAATTCTCC	
MdY3IP1-R (AD-SalI)	GTCGACTGGAGCGAATTGTAAAAC	
MdIDH1-F(Y2H Smal)	CCCGGGGCTGAGAGCGCGGCCGCAAT	
MdIDH1-R(Y2H Sal)	GTCGACAACCGCTGCAGGTTCCAGAA	
MdY3IP1-F (PGEX-BamHI)	GGATCCATGGGTTTGCAAATTCTCC	MdY3IP1 sequence for MdY3IP1-GST
MdY3IP1-R (PGEX-SalI)	GTCGACTGGAGCGAATTGTAAAAC	or MdY3IP1-His
MdDEP1-F (PET-BamHI)	GGATCCATGGCAGCGGAAGCTGAGGTTC	MdDEP1 sequence for MdDEP1-GST or
MdDEP1-R (PET-SalI)	GTCGACTATCATGCTAGCCTCTTCTTTTAG	MdDEP1-His
18S-F	TGACCGAATGAGCAAGGAAATTACT	
18S-R	TACTCAGCTTTGGCAATCCACATC	
MdDEP1-F	TGAAAGCAAGAACGGAATCTCA	
MdDEP1-R	CCTTCACTATGTTCCTCCACCAC	
MdPsaK-F	CAGGAAGTCAACGGCAGGATT	
MdPsaK -R	CAAGAACAACTCCAACCCCAAT	
MdPsaG -F	CTTCCTTGGGAGGTTTGTTTTCT	
MdPsaG -R	GGTCATTGGACTTGAGGAGGC	
MdNAC89-F	ATGGTTGTTTGCCGCCTTCG	
MdNAC89-R	GAGCATTCAACTGCCTTATCCCC	
RING/U-box superfamily protein-F	AACTGGGGAACAAAGAAGGCAT	
RING/U-box superfamily protein-R	GATGTCTCCGTGGGCATTCTCT	

MdPsaL-F	CAACCCATCAACGGAGATCCTT	
MdPsaL -R	GGGCTGACCGCTGTCCTGTAG	
MdPLP-tases-F	CTTATGCGTGGGTGAAGTGCG	
MdPLP-tases -R	TGAGACGGACATAGCGGGTTT	
MdAP2/B3-like TF -F	CCAGGTGAAGACATACGAAGCAT	qPCR primers
MdAP2/B3-like TF-R	CTTCGGCTGTCACGGTTCTC	
MdbHLH TF-F	CTGCCGAAAATGCCGTAATG	
MdbHLH TF -R	AGTTCTTATTCAACCCACTAATCAGC	
MdSAG101-F	CTGGTGACTTCTCCTCCCCTG	
MdSAG101-R	ATGGTGGTGTTTGAGTGCTTGG	
MdPsaE'-F	CAAGAGAGGCACTAAGGTGAAGATT	
MdPsaE'-R	CCACGACAGGGTAACGGGTATT	
MdPsaF'-F	CCCATTTGATTGTCAGCGGTG	
MdPsaF'-R	GTGTCGGCTTCTTATCATCCCT	
MdPsaO'-F	GATGGGGCTGGGCAGTTCCT	
MdPsaO'-R	CAATCACATTCAGATCCCTCCGC	
MdPsaD-F	GGACCCAACAACACCCTCACC	
MdPsaD-R	GCCAGTCGGCATCTCAAATATCT	
MdSAM-Mtases -F	TTCTTCTTATTCCCTTAGTTGGCTT	
MdSAM-Mtases-R	ATTACTTCTCCTGGGCTTGTGG	
MdPsaH-F	CAACCCTAGCAGCCGTTCAAC	
MdPsaH -R	GCCACCACAGCACCACTCCT	
MdSLlecRK -F	ATACTCAGAGAATTGTGGGAACGC	
MdSLlecRK-R	CTCCAATACCAAGACCCCAAAG	
MdPsbP-F	ACCAACGAGACTGCTGGAACAT	
MdPsbP -R	GAAAGCCCACCGAAGAAGAAT	qPCR primers
MdPsbR -F	GGAGGAAGGGTAAGGGAAAGG	
MdPsbR-R	GCATAGACATCACCACTTGGAGAC	
MdPsbW-F	CTGGGGCTCCATCCTCAACT	
MdPsbW -R	TAGCTGCTGCCGCCAAAGAT	
MdPsbO-F	TAGAGCCAACATCCTTCACAGTCA	
MdPsbO-R	AGAGTGTAGGTTAGGCGGGTCAT	
MdPsbS-F	CATCTTCTTTTGCCTCATCATCT	
MdPsbS -R	GTCTTCAACCTTCGGCTTCG	

MdPsbQ-F	GGGAGTTGATGGGGAGGGAT
MdPsbQ -R	CAGTTTGTTGGCAAGTTCAGTGAG
